# Artificial habitats host elevated densities of large reef-associated predators

**DOI:** 10.1371/journal.pone.0237374

**Published:** 2020-09-02

**Authors:** Avery B. Paxton, Emily A. Newton, Alyssa M. Adler, Rebecca V. Van Hoeck, Edwin S. Iversen, J. Christopher Taylor, Charles H. Peterson, Brian R. Silliman

**Affiliations:** 1 CSS-Inc., Fairfax, VA, United States of America; 2 Nicholas School of the Environment, Duke University Marine Lab, Beaufort, NC, United States of America; 3 Institute of Marine Sciences, University of North Carolina at Chapel Hill, Morehead City, NC, United States of America; 4 Department of Statistical Science, Duke University, Durham, NC, United States of America; 5 National Centers for Coastal Ocean Science, National Ocean Service, National Oceanic and Atmospheric Administration, Beaufort, NC, United States of America; University of Sydney, AUSTRALIA

## Abstract

Large predators play important ecological roles, yet many are disproportionately imperiled. In marine systems, artificial reefs are often deployed to restore degraded reefs or supplement existing reefs, but it remains unknown whether these interventions benefit large predators. Comparative field surveys of thirty artificial and natural reefs across ~200 km of the North Carolina, USA coast revealed large reef-associated predators were more dense on artificial than natural reefs. This pattern was associated with higher densities of transient predators (e.g. jacks, mackerel, barracuda, sharks) on artificial reefs, but not of resident predators (e.g., grouper, snapper). Further analyses revealed that this pattern of higher transient predator densities on artificial reefs related to reef morphology, as artificial reefs composed of ships hosted higher transient predator densities than concrete reefs. The strength of the positive association between artificial reefs and transient predators increased with a fundamental habitat trait–vertical extent. Taller artificial reefs had higher densities of transient predators, even when accounting for habitat area. A global literature review of high trophic level fishes on artificial and natural habitats suggests that the overall pattern of more predators on artificial habitats is generalizable. Together, these findings provide evidence that artificial habitats, especially those like sunken ships that provide high vertical structure, may support large predators.

## Introduction

Across terrestrial and aquatic ecosystems, large predators provide valuable services, such as enhancing ecosystem resilience and production [1, 2], facilitating ecosystem connectivity [3], and maintaining biodiversity [4]. Numbers of many large predators across different ecosystems have declined due to factors including habitat degradation, as well as unregulated fishing or hunting pressure [5]. While approaches towards and success of predator conservation remain largely context dependent, many successful conservation efforts have focused on stemming unregulated hunting and fishing [6, 7]. If overhunting and overfishing can be curtailed in certain areas, conservation strategies may then focus on providing suitable habitats for large predators [8, 9].

Ensuring that large predators have access to suitable habitats is challenging because of widespread habitat degradation in terrestrial and aquatic systems [10]. In coastal ecosystems, for example, degradation occurs in habitats ranging from coral reefs [11] and mangrove forests [12] to seagrass beds [13] and oyster reefs [14]. To supplement or restore lost habitat, artificial habitats are often installed in ecosystems [15]. Increasing numbers of artificial habitats are particularly pronounced in marine ecosystems, where these habitats are sometimes installed as artificial reefs (e.g, vessels, concrete modules) to replace degraded habitats, such as coral reefs, and are also installed to augment healthy habitats, such as rocky reefs [16]. Artificial structures can also be introduced to marine systems as offshore energy extraction facilities or unintentionally through shipwrecks.

Continued introduction of artificial habitats to marine ecosystems globally can have both positive and negative ecological effects [17, 18]. Ecological benefits include increasing connectivity among habitats [17] and potentially facilitating movement of species poleward [19], whereas negative impacts of installing artificial habitats include facilitating the spread of invasive species [20], biodiversity degradation [21], and biotic homogenization [22]. It remains debated whether artificial habitats aggregate fish from surrounding natural habitats or produce new fish biomass. The former can have substantial impacts by, for example, attracting fish from nearby natural habitats to artificial habitats intended for fishing [23]. Recent studies, however, suggest that while the degree of aggregation versus production is largely species- and system- specific, evidence for production also exists [24, 25].

Direct comparisons of fish communities occupying artificial versus natural habitats have found conflicting results. Some studies suggest that artificial habitats sometimes underperform natural habitats by hosting lower abundances and species richness [26, 27], as well as differing community structure [28, 29]. However, other studies have found the opposite for fish abundance and richness [30, 31], as well as community composition [27]. Most of the comparative studies suggesting underperformance or differing performance between artificial and natural marine habitats have focused on lower trophic levels or entire communities, rather than exclusively on higher trophic levels occupied by large predatory fishes.

Because large predators play critical roles in marine ecosystems [5], it is important to determine whether marine artificial habitats can benefit their populations. Testing whether artificial habitats support large predators requires a study system where artificial habitats have been widespread for multiple decades, where natural habitats also occur, and where large predators are commonly encountered. Here, we worked in a marine system (~200 km along the continental shelf of North Carolina, USA) where artificial habitats (artificial reefs and shipwrecks) have been widespread for multiple decades near largely non-degraded natural habitats (rocky reefs) and where large reef-associated predators, including jacks, grouper, mackerel, snapper, and sharks, are frequently encountered. We asked: do densities of large reef-associated predators differ on natural versus artificial reefs? We then investigated whether differing morphologies and vertical extents of reefs may be mechanisms behind observed patterns. We also conducted a literature review to test the generality of our findings in marine and coastal ocean systems. In our literature review, we recorded whether community metrics (abundance, density, biomass) of fishes characterized as top consumers on marine artificial habitats (e.g., artificial reefs, shipwrecks) differed from corresponding values of these metrics on natural habitats (e.g., rocky reefs, coral reefs).

## Methods

### Observational data

**Fig 1:**
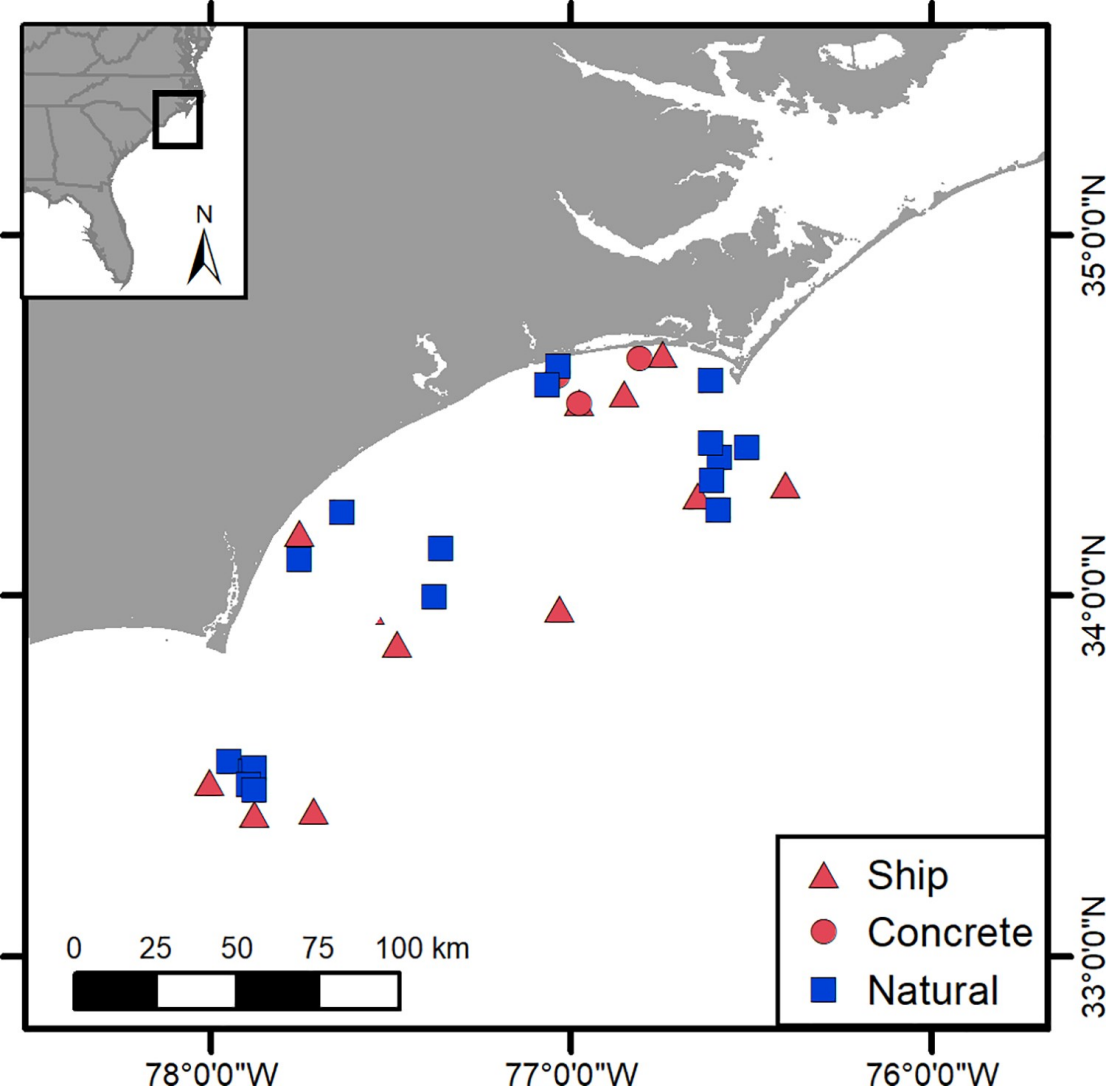
Location of study sites along the east coast of the US. Red represents fourteen artificial reefs. Red triangles are ships, and red circles are concrete artificial reefs. Blue squares represent sixteen natural rocky reefs.

We used observational data from thirty artificial and natural warm-temperate reefs to test how density of large reef-associated predators differed on artificial versus natural reefs (Fig 1). The dataset was collected in 2013–2015 using scuba-diver surveys on fourteen artificial reefs and sixteen natural reefs on the continental shelf of North Carolina, USA. Artificial reefs include ships and concrete pipes purposely deployed to enhance fish habitat, as well as historic shipwrecks [29]. Natural reefs include rocky, hard-bottom structures ranging in morphology from flat pavements and rubble fields to complex ledges [29]. The reefs range from 10–33 m deep, and all are open to fishing, including spearfishing, and diving. The area encompassed by the studied reefs is known to host a diversity of fish species, including tropical, subtropical, and temperate fishes [19, 32] Aggregations of sand tiger sharks [33] and other large reef-associated predators, including snapper, grouper, jacks, and mackerel, are also known to occur on these reefs [29, 34]).

On each of fourteen artificial reefs and sixteen rocky reefs within Onslow Bay and Long Bay, North Carolina (Fig 1), scuba-divers conducted sampling events where they visually surveyed fish along two 30-m x 4-m (120 m^2^) belt transects seasonally established along prominent reef features, following Paxton et al. [29] (S1 Table of S1 Data). If a prominent feature did not exist, the transect direction was selected from a list of randomly generated compass headings [35]. At each reef, transect location varied among seasons. Thirty-m long transects have been demonstrated to be accurate, precise, and efficient for assessing fish density on reef habitats [36], and a prior study in the same geographic area as ours found that 77% of fish were accounted for 30 m outward from reefs. On each belt transect, divers recorded the abundance of all fishes present throughout the water column, including both conspicuous (e.g., easily discernable fishes not hiding in reef structures) and cryptic (e.g., smaller fishes often hiding in reef structures) categories of reef fishes, to the lowest taxonomic level possible. In total, 236 transects were conducted, 109 on artificial reefs and 127 on natural reefs.

We calculated the density of large reef-associated predators on each 120 m^2^ transect during a sampling event. In particular, we classified fish as large reef-associated predators based on size and diet as reported in Fishbase [37] and regional studies published in the peer-reviewed literature. Following the recommendation of Heupel et al. [38] to classify marine predators by size and diet, we focused on species who can grow to 70 cm or larger and who eat other fish. We designated fish that can grow to 70 cm or larger as large predators based on our prior field surveys on offshore NC reefs, where this threshold is ‘large’ in the context of these reefs [29, 39]. In total, we classified twenty-seven species as large reef-associated predators, including species in the families Belonidae (needlefish), Carangidae (jacks), Carcharhinidae (sand bar shark), Dasyatidae (southern stingray), Haemulidae (grunts), Lutjanidae (snapper), Odontaspididae (sand tiger shark), Paralichthyidae (flounder), Rachycentridae (cobia), Rhincodontidae (nurse shark), Scombridae (mackerel), Serranidae (grouper), Sparidae (porgy), and Sphyraenidae (barracuda) (Table 1). We pooled density values for two species, barracuda (*Sphyraena barracuda*) and guachanche (*Sphyraena guanchancho*), at the genus level as *Sphyraena* sp. because similarities in appearance made it difficult for divers to distinguish them at the species level. The species that we classified as predators are likely mesopredators or top predators depending on the ecosystem specific context, as well as food web dynamics, such as whether these species exert diffuse or concentrated predation pressure [36].

**Table 1:** Observed density (# per 120 m^2^) and frequency of occurrence of large reef-associated predators on artificial versus natural reefs per 120 m^2^ transect. For each species, raw abundance over 109 transects of artificial reefs and 127 transects on natural reefs is provided, along with the frequency of occurrence (%) that corrects for differing number of transects by reef type. Mean observed density ± SE is provided. *Sphyraena* sp. represents pooled values of two species, barracuda (*Sphyraena barracuda*) and guachanche (*Sphyraena guanchancho*).

Fish species				Raw density		Frequency of occurrence (%)		Mean density	
Family	Scientific name	Common name	Residency	Artificial	Natural	Artificial	Natural	Artificial	Natural
Belonidae	*Ablennes hians*	Flat Needlefish	Transient	0	3	0.00	0.79	0.00±0.00	0.02±0.02
Carangidae	*Carangoides bartholomaei*	Yellow Jack	Transient	165	72	21.11	4.96	1.51±0.64	0.57±0.32
	*Seriola dumerili*	Greater Amberjack	Transient	640	123	65.15	27.00	5.87±1.57	0.97±0.25
	*Seriola rivoliana*	Almaco Jack	Transient	84	13	10.10	4.96	0.77±0.48	0.10±0.05
Carcharhinidae	*Carcharhinus plumbeus*	Sandbar Shark	Transient	1	0	0.93	0.00	0.01±0.01	0.00±0.00
Dasyatidae	*Dasyatis americana*	Southern Stingray	Resident	8	0	6.86	0.00	0.07±0.03	0.00±0.00
Haemulidae	*Anisotremus surinamensis*	Black Margate	Resident	19	9	4.81	2.42	0.17±0.12	0.07±0.05
Lotidae	*Brosme brosme*	Cusk	Resident	1	0	0.93	0.00	0.01±0.01	0.00±0.00
Lutjanidae	*Lutjanus campechanus*	Red Snapper	Resident	8	22	3.81	5.83	0.07±0.04	0.17±0.08
	*Lutjanus griseus*	Gray Snapper	Resident	5	0	2.83	0.00	0.05±0.03	0.00±0.00
	*Ocyurus chrysurus*	Yellowtail Snapper	Resident	0	1	0.00	0.79	0.00±0.00	0.01±0.01
Odontaspididae	*Carcharias taurus*	Sandtiger Shark	Transient	48	1	17.20	0.79	0.44±0.14	0.01±0.01
Paralichthyidae	*Paralichthys albigutta*	Gulf Flounder	Resident	11	25	9.00	9.48	0.10±0.03	0.20±0.07
	*Paralichthys dentatus*	Summer Flounder	Resident	13	9	10.10	5.83	0.12±0.04	0.07±0.03
	*Paralichthys lethostigma*	Southern Flounder	Resident	4	1	3.81	0.79	0.04±0.02	0.01±0.01
Rachycentridae	*Rachycentron canadum*	Cobia	Transient	1	0	0.93	0.00	0.01±0.01	0.00±0.00
Rajidae	*Dipturus laevis*	Barndoor Skate	Resident	0	1	0.00	0.79	0.00±0.00	0.01±0.01
Rhincodontidae	*Ginglymostoma cirratum*	Nurse Shark	Transient	1	0	0.93	0.00	0.01±0.01	0.00±0.00
Scombridae	*Euthynnus alletteratus*	Little Tunny	Transient	146	8	3.81	0.79	1.34±0.96	0.06±0.06
	*Scomberomorus cavalla*	King Mackerel	Transient	250	0	1.87	0.00	2.29±1.65	0.00±0.00
	*Scomberomorus maculatus*	Spanish Mackerel	Transient	592	2	4.81	1.60	5.43±3.83	0.02±0.01
Serranidae	*Epinephelus guttatus*	Red Hind	Resident	2	0	1.87	0.00	0.02±0.01	0.00±0.00
	*Mycteroperca interstitialis*	Yellowmouth Grouper	Resident	1	0	0.93	0.00	0.01±0.01	0.00±0.00
	*Mycteroperca microlepis*	Gag	Resident	193	197	78.69	73.97	1.77±0.35	1.55±0.26
	*Mycteroperca phenax*	Scamp	Resident	91	108	39.74	42.70	0.83±0.18	0.85±0.17
Sparidae	*Pagrus pagrus*	Red Porgy	Resident	3	0	1.87	0.00	0.03±0.02	0.00±0.00
Sphyraenidae	*Sphyraena* sp.	Barracuda	Transient	663	207	43.42	6.72	6.08±4.61	1.63±0.92

We further classified the large reef-associated predators by their residency to reefs (Table 1). We designated species that more transiently associate with reefs, such as fast-swimming, highly-mobile, schooling species, which often appear in the water column above or around reefs, as ‘transient.’ Transients included fish, such as jacks [40], mackerel [41], barracuda [42], and sharks [43]. We classified demersal species more commonly associated with the reef structure or seafloor as ‘resident.’ The residents included fish, like snapper, grouper, flounder, and rays, which often exhibit high degrees of site fidelity and residence time to natural and artificial reefs [41, 44–48]. We made this classification based on previous studies cited above detailing residency patterns for these species, as well as our ecological knowledge from extensive observations of which species associate with the reef structure versus the water-column above and because our of personal observations that water-column associated species tended to be more prevalent on artificial versus natural reefs.

### Statistical analysis

Statistical analyses were conducted in R version 3.5.3 [49]. We used generalized linear mixed models (GLMMs) [50] to model the relationship between densities of large reef-associated predators, which was the response variable, and reef and species characteristics [51] (S1 Text of S1 Data). Density values were measured as species-specific, transect-level counts observed during a single sampling event. We included reef type (artificial vs. natural) and species ‘residency’ status (transient vs. resident) and their interaction, as well as reef depth and sampling season (winter, spring, summer, fall), as fixed effects. We calculated reef depth as the mean depth recorded by a pressure transducer (Onset Hobo U20 Titanium Water Level Logger, U20-001-02-Ti) moved along the transects at a particular reef (see [29] for methods details). We included individual species and sampling event nested within reef as random effects, the latter to allow for reef-to-reef variation not described by the model’s fixed effects and for the possible correlation of predator densities on a reef’s transects visited during the same sampling event. We fit the model with the ‘glmmTMB’ package [52]. We used negative binomial models to allow for overdispersion in the counts relative to the Poisson model and the log link function. Likelihood ratio tests (LRTs) were used to assess strength of evidence in favor of associations between predator density and reef type, residency status and their interaction, as well as associations with season and reef depth.

We used GLMMs and generalized linear models (GLMs) to investigate potential mechanisms, such as reef morphology and vertical relief, behind observed patterns in predator densities by reef type. First, we examined the relationship between specific reef morphologies and predator density. We did this by refitting the GLMM model above (fixed effects: reef type x residency, depth, season; random effects: sampling event nested within reef, species), and conducting accompanying LRTs, with reef morphology replacing reef type as a fixed effect. Reef morphology is a factor containing four levels, two for each reef type (artificial–ship, artificial–concrete, natural–pavement-and-rubble, natural—ledge). Morphology levels for artificial reefs relate to characteristics, such as reef material and spatial extent, as ships are metal, isolated structures, whereas concrete structures are often dozens to hundreds of individual structures dispersed across a broader area. Morphology levels for natural reefs also relate to reef characteristics, as pavement-and-rubble reefs are often low-lying, ephemeral habitats that can be covered and uncovered by sand, whereas ledges are more pronounced, less ephemeral habitats.

Second, we examined the relationship between reef vertical relief and predator density. We calculated vertical relief as the difference between the deepest and shallowest point on each reef transect, as recorded by a pressure transducer (Onset Hobo U20 Titanium Water Level Logger, U20-001-02-Ti) moved continuously along the transect (see [29] for methods details). Using the maximum vertical relief (m) values for each reef across sampling events and transects, we fit a negative-binomial model for total predator density to: (1) the full data set, (2) the subset of transient predators on artificial reefs, and (3) the subset of data corresponding to transient predators on artificial reefs that were ships with known length using the ‘MASS’ package [53]. This third model allowed us to isolate the effect of reef size from the relationship between transient predator density and reef relief. We were able to do this only for the subset of artificial reefs that were ships of known lengths prior to sinking. We do not know the habitat size of the remaining natural and artificial reefs, as most remain unmapped. We extracted information on ship lengths from historical information for shipwrecks and from the NC Division of Marine Fisheries for artificial reefs. We then scaled transient predator densities on these ships by the ship length. We converted these normalized predator values to integers and used them as the response variable regressed against the predictor variable vertical relief in our third GLM. For all GLMs, we conducted LRTs between the full model and the reduced model (e.g., model without vertical relief) to determine the effect of vertical relief on predator density, transient predator density, and scaled transient predator density, respectively.

### Literature review

To test whether patterns observed in diver data were generalizable, we conducted a literature search and accompanying analysis of reef-associated predators on artificial versus natural habitats in marine and coastal ocean environments (Table 2). We set our literature search query *a priori*, and conducted our literature search on 10 October 2018 using Web of Science. Our search included artificial reefs, oil platforms, and shipwrecks as artificial habitats and coral reefs and rocky reefs as natural habitats. We used the advanced search function with Boolean logic and the following search query: (artificial reef OR artificial habitat OR shipwreck OR oil platform OR oil rig) AND (natural reef OR rocky reef OR coral reef) AND (fish community OR fish assemblage). This search yielded 479 potentially relevant studies. We imported titles and abstracts from all 479 studies into Colandr [54] and screened each using specified inclusion criteria (Table 2). Of the 479 studies, 93 were selected as potentially relevant and included in a subsequent full-text screening based on the same criteria. Of the 93 studies for which full-text screening was conducted, fourteen met our criteria and were retained for data extraction.

**Table 2:** Study inclusion components and criteria for literature review of fish occurrence on artificial versus natural reefs.

Inclusion component	Inclusion criteria
Subject(s)	Fish species that are large reef-associated predators, as assigned by trophic level ≥ 4.4 in Fishbase
Comparator	Natural habitats versus artificial habitats in marine or coastal ocean environments. Natural habitats included rocky reefs and coral reefs. Artificial habitats included artificial reefs, oil platforms, and shipwrecks. Estuarine habitats (salt marshes, seagrasses, mangroves), shoreline habitats (breakwaters, piers, ports, marinas, jetties), and habitats within the Mediterranean Sea were excluded.
Response(s)	Abundance, biomass, density, or occurrence of fishes on natural versus artificial habitats.
Study type	Primary research studies reporting field observations of fish assemblages or communities on artificial and natural reefs from visual surveys (e.g., diver surveys, video surveys). Meta-analyses, reviews, or studies with indirect observations (e.g., stable isotopes, gut contents, fisheries catch) were excluded.

Data on how many fishes were present on each reef type were extracted from the fourteen studies. For each study, we extracted the scientific name of each fish species for which data existed. We then queried Fishbase [37] for the trophic level of each species. Trophic levels are assigned based on diet studies, natural history, and other related information [37] (S2 Text of S1 Data). We focused on fishes with trophic levels of 4.4 and 4.5 because these are often reef-associated predators, including some top predators and some mesopredators. There were forty-two records of fishes with trophic levels 4.4 and 4.5 with reported abundance, density, or biomass on artificial versus natural marine habitats. We further refined this list by examining prey items and body sizes to keep only those fish regarded as reef-associated predators. For example, we removed tomtate (*Haemuleon aurolineatum)* which have a high trophic level on Fishbase but are not reef-associated predators in many locations. Our refined list came from six unique studies, as eight of the original fourteen studies did not include large predatory fish, as defined on the basis of trophic level (S5 Table of S1 Data). The reefs in these studies did not have protection status, except for one where shrimp bottom trawling was prohibited on artificial reefs [55]. The final six studies collectively reported on eighteen predatory fish species (S6 Table of S1 Data); for each, we verified status as reef-associated predators and classified each as either resident or transient based on level of reef association. Because several studies reported on the same species of fish as other studies, our final extracted data included 26 reports of these 18 fish species of large predatory fish. Our list is likely conservative or restricted because fishes with trophic levels < 4.4 were not included yet may fill roles as large reef-associated predators.

For each reef-associated predator species from each study, we extracted metadata for the study, including the title, date, authors, geographic location, survey method (e.g., scuba belt transect vs. remotely-operated vehicle survey), artificial reef type (e.g., vessel vs. concrete), and reef depth. We then extracted the abundance, density, or biomass of each species from each study on artificial versus natural habitats. We also extracted any available measures of precision, such as standard error, standard deviation, or *p*-values and test statistics from which precision metrics can be calculated. Because several studies reported measures of precision whereas others did not report these values and because the measurements (e.g., abundance, density, biomass) differed among studies, we could not carry out a formal meta-analysis to estimate a global effect. Since a formal, precision-based weighting was not possible, we treated all studies with equal weight and because the abundance metrics varied by study, we summarized the data using a binary variable computing whether the species was more dense on artificial than natural habitats (= 1) versus not (= 0).

We estimated the log odds of reef-associated predators exhibiting higher values (e.g., abundance, density, or biomass) on artificial reefs than natural reefs by fitting a logit link binomial GLMM [50] with the ‘glmmADMB’ package [56]. We did not include residency status in the model because the sample size was too low. Because the data were extracted from six studies using different survey methods across multiple geographic areas, we included study as a random effect. The model’s only fixed effect was its intercept; it is the quantity of interest because its interpretation is as the log odds of reef-associated predators being present on artificial habitats in higher values than on natural reefs. We used a one-sided test of the hypothesis that predators are equally dense on the two reef types versus more dense on artificial reefs because we turned to the literature review to validate our findings from the observational field data that reef-associated predators would be present on artificial reefs at higher values than on natural reefs. We computed point and 95% interval estimates of the odds of predator abundance being greater on artificial reefs.

## Results

### Observational data

Comparative field surveys of fourteen artificial reefs and sixteen natural reefs across ~200 km of the North Carolina, USA coast revealed that large reef-associated predators were 4.3 times more dense on artificial than natural reefs. Mean observed predator density across species per sampling transect conducted on artificial reefs was 1.00 ± 0.24 SE, whereas mean density across species on natural reefs was 0.23 ± 0.04 SE. This pattern is based on relative densities of large fish predators observed during diver-conducted visual surveys (236 transects) conducted seasonally on the reefs in 2013–2015 (S1 Table of S1 Data; Fig 1). The large fish predators include twenty-seven species defined on the basis of their size and diet and designated as either transient or resident by its reef association (Table 1). Of these twenty-seven species, all but eleven were rare.

**Fig 2:**
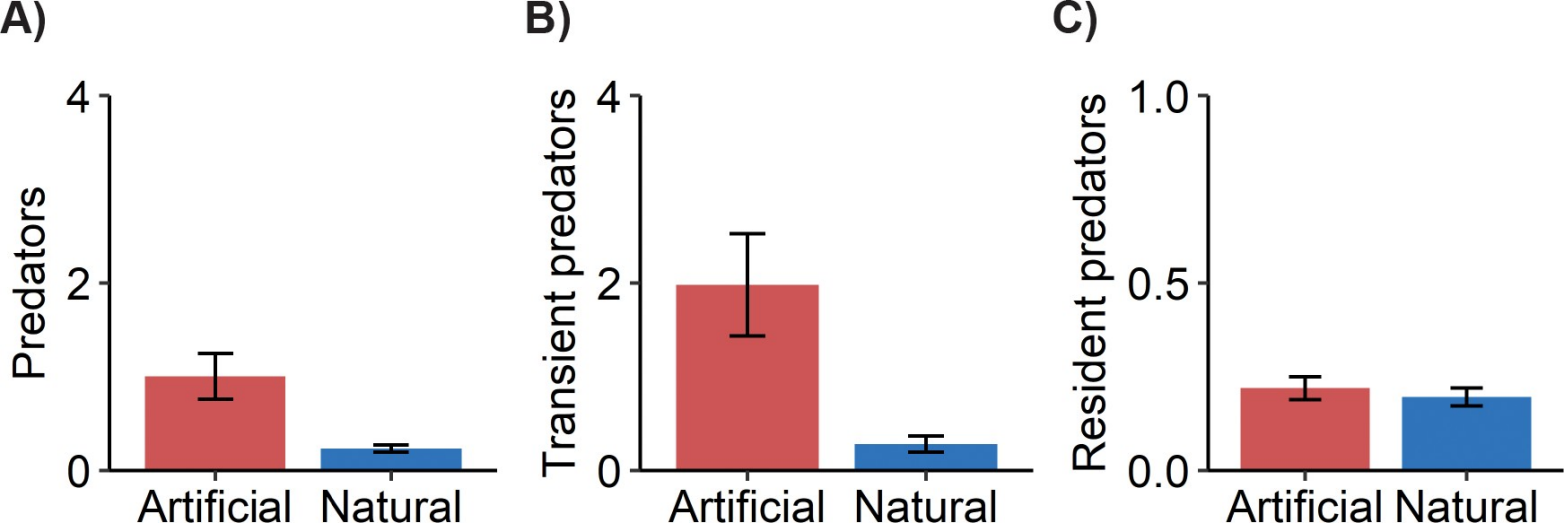
Mean observed density (± SE) per transect of predators on artificial reefs (red) versus natural reefs (blue) for A) all predators, B) transient predators, and C) resident predators. These values represent aggregated species-specific densities. N = 109 transects for artificial reefs and 127 transects for natural reefs.

When we examined patterns of predator density, we found that, although large reef-associated predators were more dense on artificial reefs than nearby natural reefs (Fig 2A; Table 1; S1 Fig and S2 Table of S1 Data; LRT reef type, NB1, χ^2^ = 44.38, *p* < 0.0001), this pattern was driven by higher densities of transient predators on artificial reefs (Fig 2B; S3 Table; LRT reef type x residency, NB1, χ^2^ = 44.42, *p* < 0.0001). These more transient predators, which often associate with reef structures and the water column above, include jacks (e.g., greater amberjack, almaco jack, yellow jack), mackerel (e.g., Spanish mackerel, king mackerel, little tunny), and sharks (e.g., sand tiger sharks). Our model estimated that transient predators were five times as dense on artificial than natural reefs, as the estimated mean transient predator density per transect was 0.31 ± 0.15 on artificial reefs and 0.08 ± 0.04 on natural reefs at the average reef depth (20 m) during both the summer and fall seasons (S3 Table of S1 Data). While there was a low frequency of occurrence for most of the transient predators, when they were observed, they were nearly always observed on artificial reefs (Table 1; S1 Fig of S1 Data). For example, king mackerel were only observed on artificial reefs but with a low frequency (1.9%). Three species, nurse shark, sandbar shark, and cobia were only observed once throughout the study but always on artificial reefs (0.9%). Spanish mackerel and little tunny were observed < 5% of the time on artificial reefs. Some of the transient species were more frequently observed on artificial reefs, including almaco jack (10.1%), sand tiger sharks (17.2%), yellow jack (21.1%), barracuda (43.4%), and greater amberjack (65.2%).

In contrast, predators who are more resident to reefs, often residing closer to the seafloor, did not follow this pattern (Fig 2C; Table 1; S2 Fig and S3 Table of S1 Data). The more resident species, including grouper (e.g., gag, scamp) and snapper (e.g., red snapper), displayed similar densities on artificial and natural reefs. Our model estimated that mean resident predator density was 0.18 ± 0.08 and 0.14 ± 0.06 per transect on artificial and natural reefs, respectively, at the average reef depth (20 m) during the summer and fall (S3 Table of S1 Data). Some resident predators exhibited high frequencies of occurrence on both reef types (Table 1; S2 Fig of S1 Data). For example, gag and scamp grouper were observed >75% and ~ 40% of the time on both reef types, respectively. Other species, such as red snapper and black margate, were observed less frequently on both reef types (<10%). Several rare species, such as the barndoor skate and yellowtail snapper, were each observed once but only on natural reefs (0.8%), whereas one rare species, the southern stingray, did not adhere to the pattern and was exclusively observed on artificial reefs.

The model that we used to estimate the effect of reef type and fish residency on predator density accounted for depth and season as fixed effects and reef-to-reef and species-to-species variation as random effects. Depth related to the density of predators, with deeper reefs generally hosting higher predator densities (S2 and S3 Tables of S1 Data; LRT depth, NB1, χ^2^ = 11.15, *p* < 0.001). Season did not significantly relate to predator densities, although reefs sampled during summer and fall seasons tended to have higher predator densities than reefs sampled in spring and winter (S3 Fig; S2 and S3 Tables of S1 Data; LRT season, NB1, χ^2^ = 2.68, *p* = 0.44). The amount of variation in random effects accounted for by reefs (SD = 0.06) and sampling events (SD = 0.15) is smaller than that of species (SD = 2.32; S2 Table of S1 Data). This suggests that there are species-specific preferences. Including the predictor variable for species residency in the model reduced species variation compared to excluding this term from the model. The additional species-to-species variation unaccounted for by the residency predictor variable likely stems from differences in other species traits, such as size and schooling behavior.

**Fig 3:**
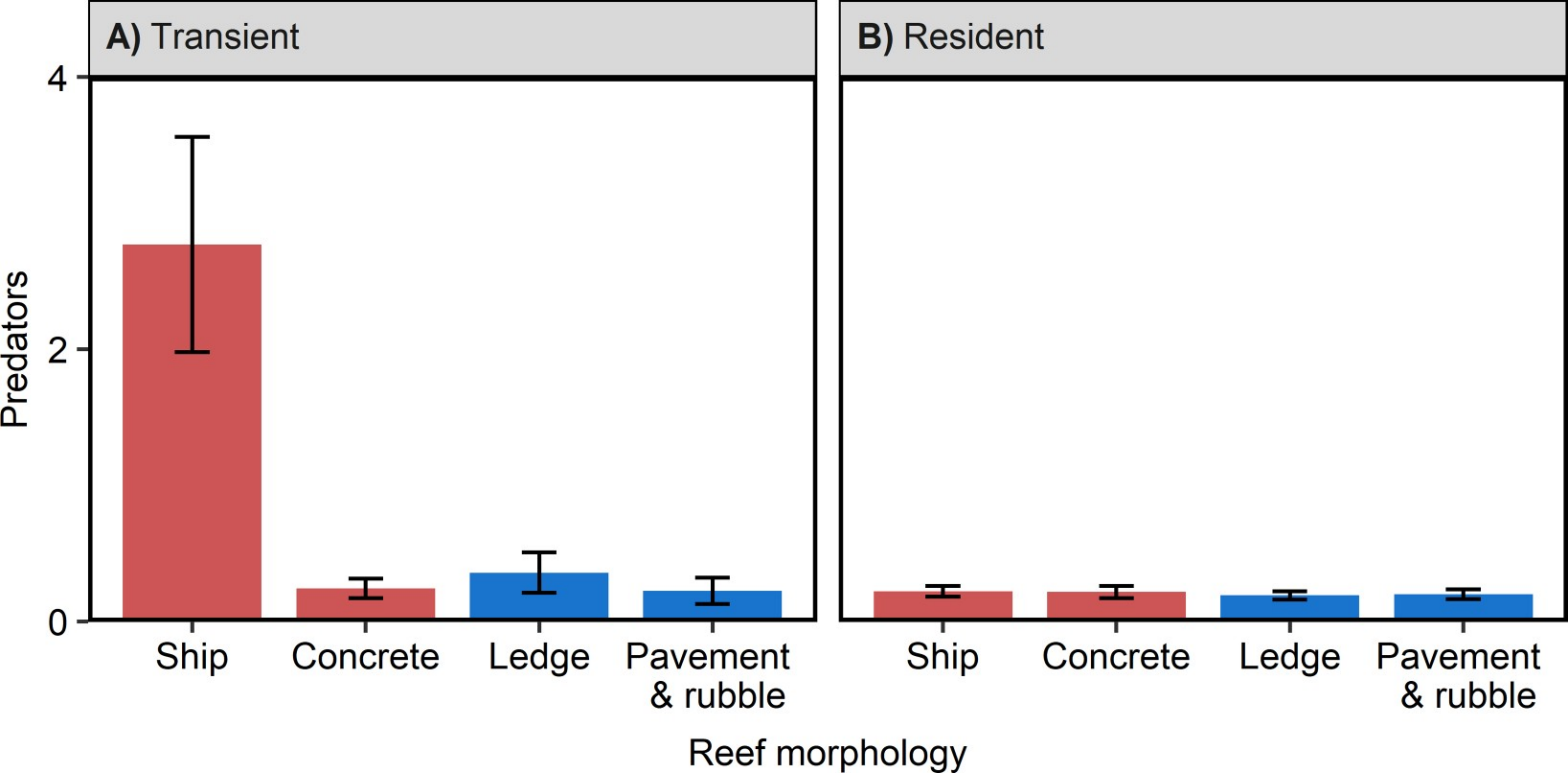
Mean observed predator density (± SE) per transect on artificial reefs (red) versus natural reefs (blue) by reef morphology for A) transient predators and B) resident predators. These values represent combined species-specific densities. N = 109 transects for artificial reefs and 127 transects for natural reefs.

**Fig 4:**
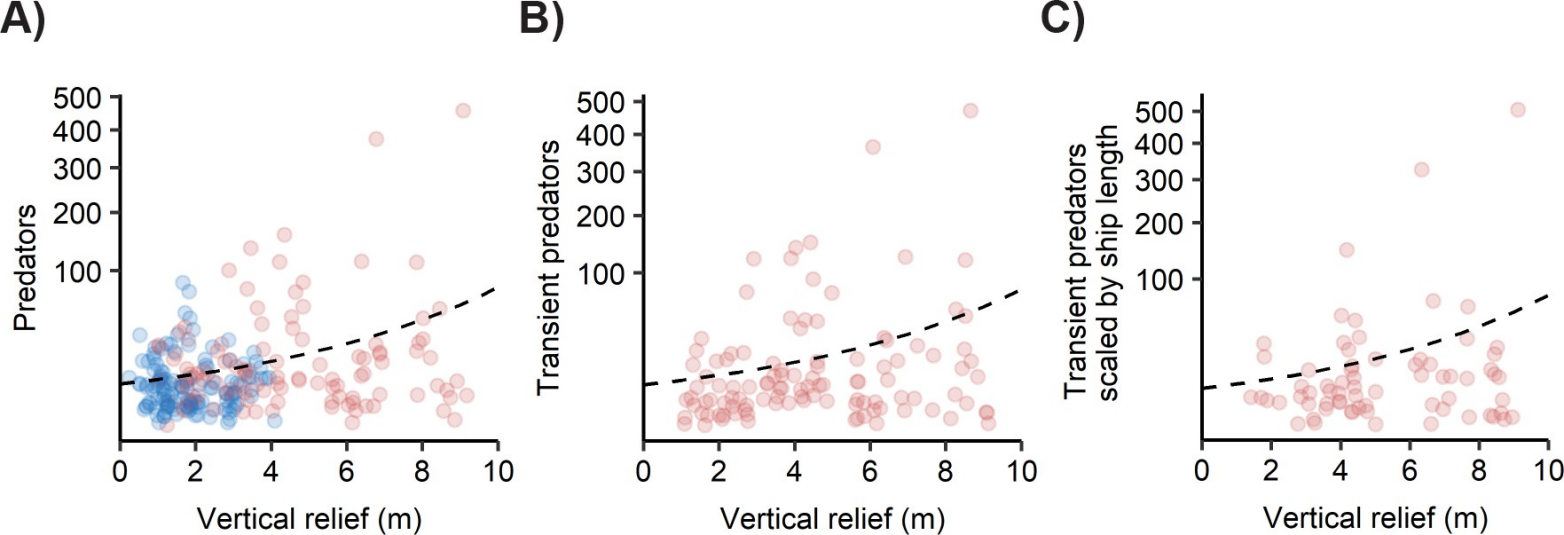
Association between vertical relief of reefs and predator density. A) Density of all predators (transient and resident) on artificial reefs (red) and natural reefs (blue). N = 109 artificial reef transects and 127 natural reef transects. B) Density of transient predators on artificial reefs. N = 109 artificial reef transects. C) Density of transient predators scaled by reef length for ship-type artificial reefs. N = 69 artificial reef transects on ships of known lengths. Black dashed lines are predicted fit of the GLMs.

Further analyses investigating potential mechanisms that may explain the pattern of elevated predator densities on artificial reefs revealed that reef morphology and reef height were associated with predator densities. Including reef morphology in the model instead of reef type improved the model fit (ΔAIC = 15.7) and suggested that higher densities of transient predators on artificial reefs were largely driven by ships (Fig 3; S2 Table of S1 Data LRT reef morphology x residency, NB1, χ^2^ = 68.14, *p* < 0.0001). The model estimated that mean transient predator density per transect was over twice as high on ships (0.37 ± 0.18) than on concrete (0.16 ± 0.09) at the average reef depth (20 m) during the summer (S4 Table of S1 Data). The observed mean transient predator densities were even higher–eleven times greater–on ships (2.77 ± 0.79 SE) than concrete artificial reefs (0.24 ± 0.07 SE) (S4 Table of S1 Data). Additionally, vertical relief of reefs positively correlated with pooled transient and resident predator densities from both artificial and natural reefs (Fig 4A; GLM NB, LRT, χ^2^ = 44.49, *p* < 0.0001). This positive effect of artificial reef relief held for transient predators on artificial reefs (Fig 4B; GLM NB, LRT, χ^2^ = 10.33, *p* < 0.01). This pattern was also preserved across a subset of artificial reefs for which we could normalize transient predator density by habitat area (Fig 4C; GLM NB, LRT, χ^2^ = 6.35, *p* = 0.01). The subset of data that we normalized was for artificial reefs that were ships of known lengths. For these ships, we used their length (length prior to sinking) as an estimate of habitat area.

### Literature review

**Fig 5:**
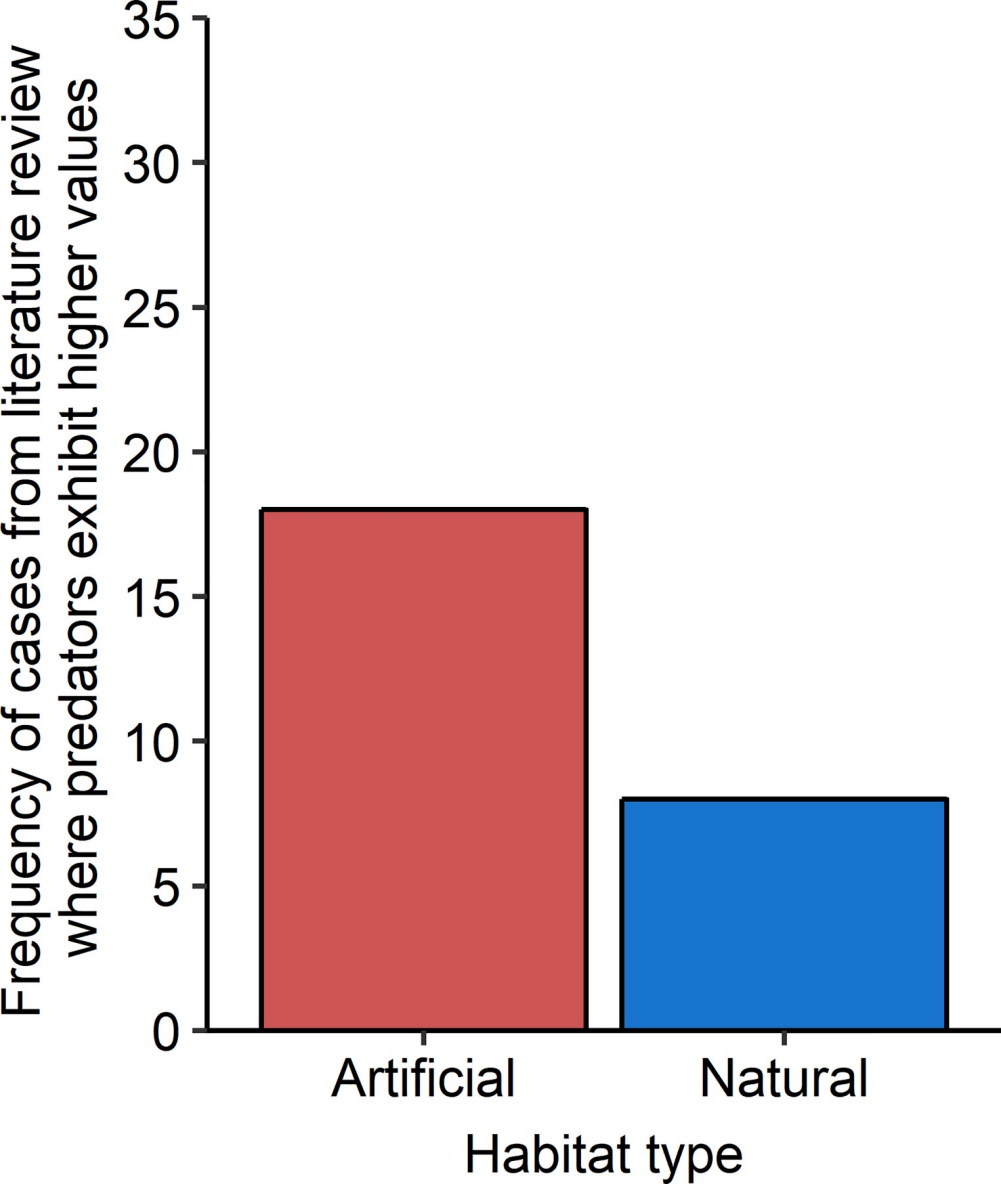
Frequency of cases from literature synthesis where fishes classified as large predators exhibited higher values (abundance, biomass, or density) on artificial habitats versus natural habitats. N = 26.

We documented twenty-six cases (reports or instances of a species within a study) in which values of large reef-associated predatory fishes were reported on both artificial and natural habitats (S5, S6 Tables of S1 Data). These twenty-six cases stemmed from six studies and represented eighteen species. Of these twenty-six cases, including observations in North America, South America, and Africa, the reef-associated predators exhibited higher values on artificial reefs in eighteen cases (69%) and on natural reefs in eight cases (31%; Fig 5). Predator residency status did not affect the outcome, as for both residents and transients, there were nine cases of higher values on artificial reefs and four on natural reefs. When we explicitly tested the odds of large reef-associated predators occurring in higher values on artificial versus natural habitats, our model revealed large predators were significantly more likely to occur in higher abundance, density, or biomass on artificial habitats compared to natural habitats (one-sided *p* = 0.03). Specifically, on the basis of this analysis, we estimate that the odds of large predator species being more abundant on an artificial than natural reef is 2.25 (95% confidence interval 0.98, 5.13). Study-to-study variation in this quantity was estimated to be small (SD = 0.04).

## Discussion

Extensive scuba-diver surveys of thirty reefs across ~200 km of the continental shelf of coastal North Carolina revealed that large reef-associated predators were more dense on artificial reefs than nearby natural reefs and that this effect was explained by transient species, such as jacks, mackerel, and sharks. Further, this pattern of higher predator density on artificial reefs was associated with reef morphology and vertical relief, as ship-type artificial reefs hosted more transient predators than concrete artificial reefs, and higher relief reefs hosted more transient predators, even when controlling for basal area. When we tested the generality of our finding of higher predator density on artificial reefs using previous studies, we found that large reef-associated predators tend to be more abundant on artificial habitats compared to natural habitats. Based on our literature review, we estimate that the odds of artificial habitats across global marine ecosystems supporting higher abundance, density, or biomass of top consumers were over two times greater than for natural habitats. These findings provide evidence that artificial reefs, and more broadly marine artificial habitats, may have important ecological functions, including the support of large reef-associated predators, which are critical components of healthy ecosystems.

We propose two explanations for why transient predators may occur in higher densities on artificial than natural reefs. Both explanations relate the positive effect of artificial reefs on transient predator density to morphology and vertical extent of habitat, as artificial reefs are nearly three times as structurally complex (rugosity and vertical relief) as nearby reefs in our study system [29]. First, transient predators often move among habitats to find prey, so they could occur in higher numbers on artificial reefs, especially ships, simply because these vertically-extensive reefs can host high abundances of prey. Many of the transient species that we observed consume small baitfish, which have been documented to occur in high abundances on artificial reefs of various structural complexities [29, 50], supporting this hypothesis. Rigorously testing this hypothesis is an avenue for future research. Second, transient predators moving from reef to reef may stop over on artificial reefs because certain artificial reefs, such as ships, are taller [29] and therefore potentially easy to distinguish from surrounding sand. In contrast, for resident predators, such as grouper and snapper, our field surveys indicate that they have similar densities on artificial and natural reefs. We do acknowledge that other habitat measures, such as area (horizontal size) or volume, could also relate to predator density.

In addition to reef morphology and vertical relief, several other reasons may explain why artificial reefs host higher densities of predators in general. First, artificial habitats may augment the capabilities of existing habitats to support large predators. For example, artificial habitats installed as replacements for degraded habitats may replace functions formerly associated with once healthy habitats. Similarly, artificial habitats deployed in locations with healthy, natural habitats may supplement, rather than replace, their function for large predators. Second, in areas that are habitat limited, artificial habitats may form otherwise unavailable sources of habitat capable of supporting large consumers. Specifically, in North Carolina, where our comparative field studies were conducted, differences in habitat availability may relate to why transient predators, but not resident predators, were more abundant on artificial than natural habitats. In this system, natural habitats are often extensive networks of ledges and flat pavements that are not degraded, so artificial habitats of both ship and concrete morphologies supplement existing functions of healthy rocky reefs. The artificial habitats of ships or fields of concrete structures are often isolated ‘islands’ of habitats, whereas the natural rocky reefs are more continuous [29].

The possibility remains that large predators are less likely to be detected on more extensive rocky reefs than on the more island-like artificial habitats simply based upon encounter rates in surveys relative to the overall area of natural reefs. Acoustic telemetry of large predators could help address this by investigating spatial movements and habitat use questions. Comparisons of predator density across the entire area of habitats, rather than standardized sampling transects as conducted here, would also help disentangle whether habitat area may drive the patterns in predator densities that we observed, as would examining reef spatial distributions and proximity to one another. Future research should determine whether large reef-associated predators receive net energetic or fitness benefits from artificial reefs through foraging or whether these large predators are merely resting on the reefs; answering this question will help determine whether artificial habitats provide an aggregation or production function for these fishes. Moreover, while the studied reefs are all open to fishing, we do not know whether differential fishing pressure among sites may relate to our findings. In particular, spearfishing is allowed on these reefs and may result in fish being more wary around our divers conducting fish transects and less likely to be detected on the transects [57].

While there are multiple approaches for the management of large marine predators, our findings initially indicate that strategic installation of artificial habitats may also be a potential tactic for managing these predators; however, this would not be a sound approach if artificial reef aggregate rather than produce predators. Based on the findings of our observational study, we propose that this method would be best suited for transient or mobile species, like jacks and barracuda, which move locally among habitats [40, 42]. This approach may also be suitable for more highly-migratory or mobile species, such as sharks, that move across multiple habitats and geographic areas during migration [3, 58]. We caution that we do not know whether artificial habitats simply aggregate large reef-associated predators or facilitate the production of these predators [23]. The broader answer to this question remains controversial, and there is likely a mixture of aggregation and production across trophic levels that occurs on marine artificial habitats [25]. If, however, artificial habitats simply aggregate predators, then using artificial habitats for predator conservation could have negative effects. For example, if predators are attracted from nearby natural habitats, then the consumptive pressure of the predators could have substantial impacts. Additionally, aggregating predators could make predator exploitation easier, possibly negating benefits of artificial reefs. These are concerns that must be addressed before artificial habitats are used to support predators. One step towards resolving these concern is testing whether predators exhibit active foraging behavior or reproductive behavior on artificial habitats that could indicate production rather than aggregation.

With these uncertainties in mind, we cannot advocate for the widespread and rapid introduction of artificial habitats globally in marine ecosystems. Instead, we suggest that in areas that may be habitat limited and also historically hosted or currently host populations of large predators that marine artificial habitats might assist predators and in such a way that these artificial habitats may facilitate conservation and management objectives.

## Supporting information

S1 Data (DOCX)

S1 File (ZIP)
