# Supplementary material for: Artificial habitats host elevated densities of large reef-associated predators
Source: PLoS One. 2020 Sep 2;15(9):e0237374. doi: 10.1371/journal.pone.0237374 (PMC7467309; doi:10.1371/journal.pone.0237374)
Supplement: S1 Data — (DOCX) [file pone.0237374.s001.docx]

**Title**: Artificial habitats host elevated densities of large reef-associated predators

**Authors:** Avery B. Paxton,^1 ,^* Emily A. Newton,^2^ Alyssa M. Adler,^2^ Rebecca V. Van Hoeck,^2^ Edwin S. Iversen Jr.,^3^ J. Christopher Taylor,^4^ Charles H. Peterson,^2^ and Brian R. Silliman^5^

**Supporting Information**


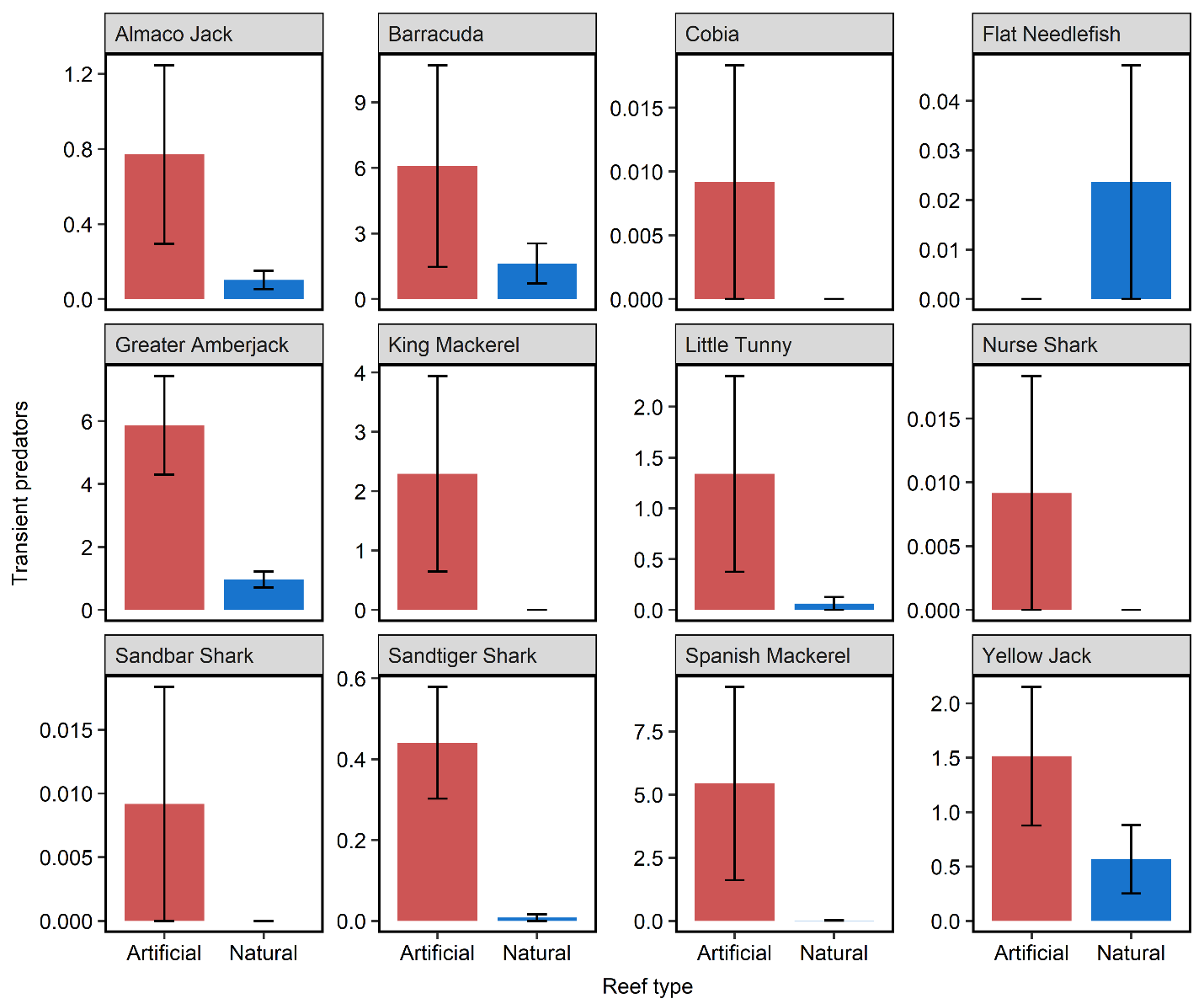


**Figure S1:** Mean observed density (± SE) per transect of transient predators on artificial reefs (red) versus natural reefs (blue) by species. N = 109 transects for artificial reefs and 127 transects for natural reefs.

**
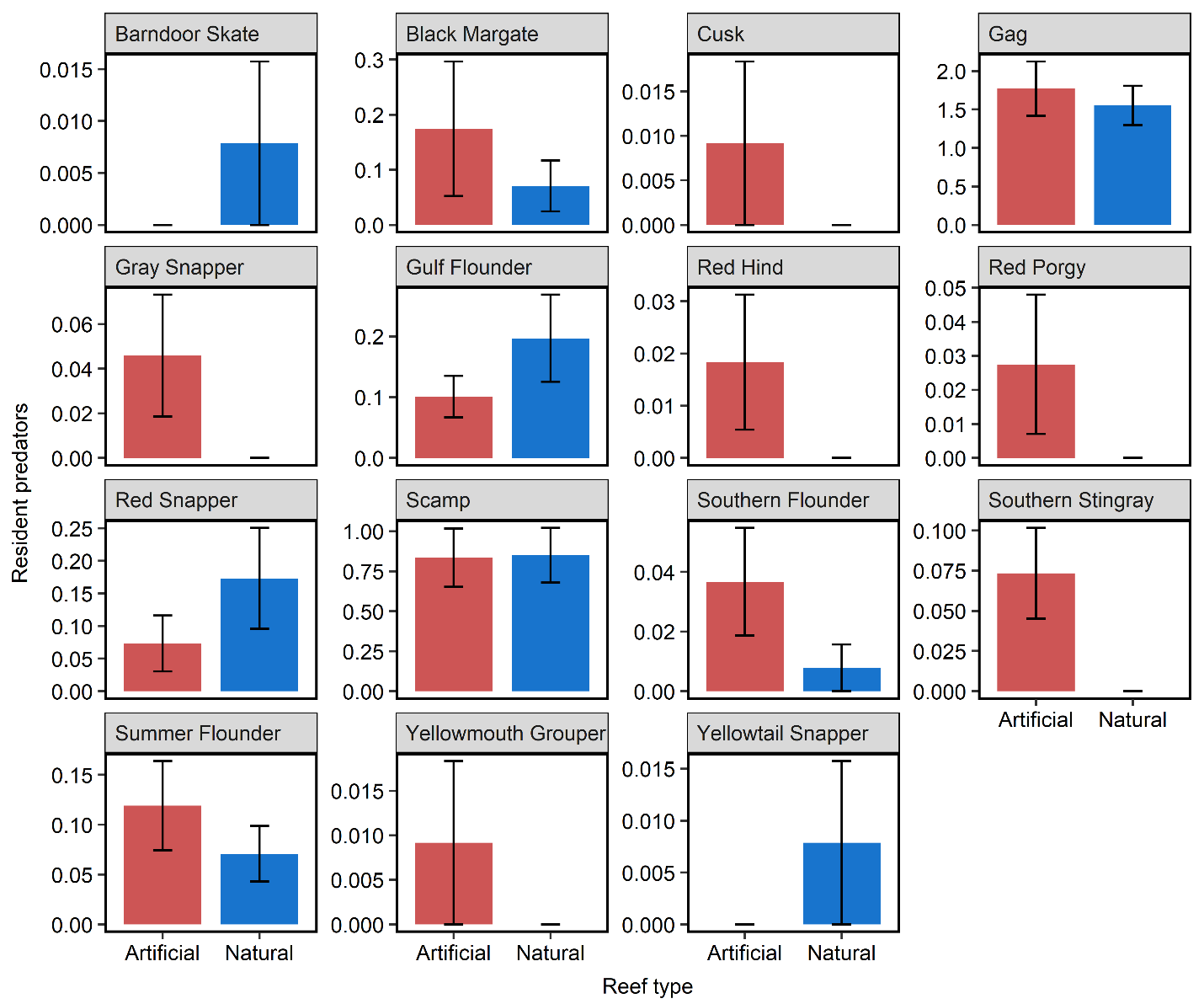
**

**Figure S2:** Mean observed density (± SE) per transect of resident predators on artificial reefs (red) versus natural reefs (blue) by species. N = 109 transects for artificial reefs and 127 transects for natural reefs.


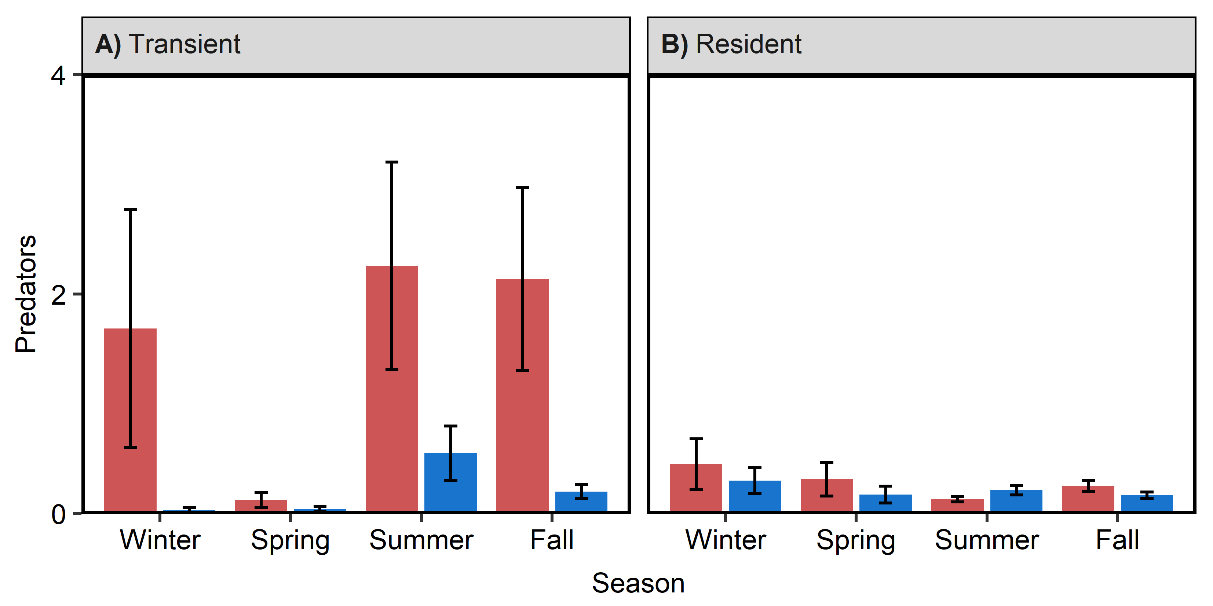


**Figure S3:** Mean observed predator density (± SE) per transect on artificial reefs (red) versus natural reefs (blue) by sampling season for A) transient predators and B) resident predators. N = 109 transects for artificial reefs and 127 transects for natural reefs.

**Text S1: Model fitting procedures for GLMMs with predator density and reef type and species residency**

For the GLMMs used to model the relationship between abundances of large reef-associated predators and reef and species characteristics, we fit the model with the following fixed and random effects. The model included fixed effects for reef type (artificial vs. natural), species residency (resident vs. transient), and their interaction, as well as for sampling season and reef depth. Random effects for individual species were included in the model. We also included sampling event nested within reef as random effects to allow for reef-to-reef variation not described by the model’s fixed effects and for the possible correlation of predator densities on a reef’s transects visited during the same sampling event. Sampling events often contained multiple transects. Sampling seasons were defined as spring (March – May), summer (June – August), fall (September – November), and winter (December – February). Reef depth was the average depth across transects at each site as calculated from the pressure transducer measurements. The response variable was predator density measured as species-specific, transect-level counts observed on a single sampling event. We fit candidate GLMMs with different error distributions appropriate for count data. First, we fit a model with a Poisson error distribution with a log-link function in the ‘glmmTMB’ package [1] using Laplace approximations for parameter estimation. We tested for overdispersion, and our data were over-dispersed because the ratio of the sum of squared Pearson residuals and the residual degrees of freedom was >1 [2] . Because our count data showed evidence of over-dispersion relative to the Poisson model, we then fit two additional models appropriate for overdispersed count data: negative binomial where variance = µ(1+ µ/*k*) (NB1; [3]) and negative binomial where variance = ϕµ (NB2; [3]), each with a log-link function in the ‘glmmTMB’ package [1] using Laplace approximations for parameter estimation. AIC comparison of the negative-binomial models indicated that the fit was better with the NB1 error distribution, so we used that model as our final model.

To determine whether there was an effect of reef type and residency on reef-associated predator abundance, we conducted a likelihood ratio tests (LRTs) with a full model and a reduced model that did not contain the predictor variable reef type, a full model and a reduced model that did not contain the predictor variable residency, and a full model and a reduced model that did not contain the reef type, residency, or their interaction. We also conducted LRTs comparing full models and reduced models for season and depth. We obtained model predictions of predator densities for each combination of reef type and species residency using the ‘predict’ function for reefs at 10 m, 20 m (mean study depth), and 30 m during each sampling season. To graphically assess model fit, we plotted the estimated probability distribution with the observed values to ensure that the observed values appeared typical of the predicted distribution.

References:

1. Brooks ME, Kristensen KK, van Benthem KJ, Magnusson A, Berg CW, Nielsen A, et al. glmmTMB balances speed and flexibility among packages for zero-inflated generalized linear mixed modeling. The R Journal. 2017;9(2):378-400.

2. Zuur AF, Ieno EN, Walker NJ, Saveliev AA, Smith GM. Mixed effects models and extensions in ecology with R. New York, NY: Springer Science + Business Media LLC; 2009.

3. Hilbe JM. Negative binomial regression. Cambridge, UK: Cambridge University Press; 2011.

**Text S2: Trophic level explanation.**

Trophic levels in Fishbase [1] range from 1 to 4.5, representing the lowest and highest trophic levels, respectively. Trophic level is calculated as:

$$Trophic level=1+mean trophic level of prey$$

Therefore, a trophic level of 1 represents phytoplankton (e.g., trophic level = 1 + 0 prey = 1), and a trophic level of 2 represents herbivorous zooplankton (e.g., trophic level = 1 + 1 prey = 2). For an animal that consumes 50% phytoplankton and 50% herbivorous zooplankton, the trophic level would then be 2.5 (e.g., trophic level = 1 + ((1 for phytoplankton + 2 for zooplankton) / 2) = 2.5) [1]. We focused on fishes with trophic levels of 4.4 and 4.5 because these are often reef-associated predators, including some top predators and some mesopredators.

References:

1. Froese R, Pauly D. FishBase. 2018. http://www.fishbase.org.

**Table S1: Descriptions of thirty reefs where diver surveys were conducted.** Coordinates are provided to the nearest tenth of a degree for latitude (Lat) and longitude (Lon). Reef depth (dep) is also provided. Date indicates month, day, and year (month/day/year) of replicate transects (1-2 transects per sampling date). Dates were used to obtain seasons as spring (March – May), summer (June – August), fall (September – November), and winter (December – February) Coordinates of a previously unknown wreck are purposely excluded.

| **Reef**  **name** | **Reef**  **type** | **Reef**  **morphology** | **Lat**  **(dd)** | **Lon**  **(dd)** | **Dep (m)** | **Date** |
| --- | --- | --- | --- | --- | --- | --- |
| Theodore Parker (AR-315) | Artificial | Ship | 34.7 | -76.7 | 10.2 | 7/8/2013, 10/29/13, 7/29/14, 10/2/14, 10/20/14 |
| Atlantic Beach Bridge (AR-320) | Artificial | Concrete | 34.7 | -76.8 | 15.2 | 10/31/2013, 7/29/14, 9/29/14, 10/20/14, 5/19/15 |
| Concrete Pipes, 2007 (AR-342) | Artificial | Concrete | 34.6 | -77.0 | 16.1 | 9/6/2013, 6/3/14, 8/20/14, 10/21/14, 5/19/15 |
| USS Indra (AR-330) | Artificial | Ship | 34.6 | -76.9 | 15.0 | 6/9/2014, 8/21/14, 5/18/15 |
| Titan (AR-345) | Artificial | Ship | 34.5 | -77.0 | 16.6 | 7/18/2013, 10/28/13, 6/2/14, 10/2/14, 10/21/14, 5/19/15 |
| Concrete Pipes, 2006 (AR-345) | Artificial | Concrete | 34.5 | -77.0 | 18.7 | 7/18/2013, 10/28/13, 6/2/14, 10/2/14, 10/21/14, 5/19/15 |
| Yard Oiler FS-26 (AR-300) | Artificial | Ship | 34.3 | -76.4 | 26.4 | 6/17/14 |
| Spar (AR-305) | Artificial | Ship | 34.3 | -76.6 | 28.3 | 8/30/2013, 6/16/14, 10/10/14 |
| *Alexander Ramsey* | Artificial | Ship | 34.2 | -77.8 | 12.0 | 9/19/2013, 7/1/14, 9/5/14, 11/13/14 |
| *Cassimir* | Artificial | Ship | 34.0 | -77.0 | 32.6 | 9/4/2014, 11/5/14 |
| *John D. Gill* | Artificial | Ship | 33.9 | -77.5 | 25.1 | 9/24/2013, 6/24/14, 8/7/14, 11/13/14 |
| *Raritan* | Artificial | Ship | 33.5 | -77.9 | 21.5 | 9/25/2013, 6/25/14, 9/11/14, 12/18/14 |
| *City of Houston* | Artificial | Ship | 33.4 | -77.7 | 28.0 | 9/25/2013, 6/25/14, 9/11/14 |
| Unknown Wreck | Artificial | Ship | ----- | ----- | 29.0 | 12/4/2013, 6/25/14, 9/11/14 |
| Keypost Rock | Natural | Pavement & rubble | 34.6 | -77.0 | 15.0 | 10/24/14 |
| Barge Rock | Natural | Ledge | 34.6 | -76.6 | 16.1 | 8/19/2013, 7/30/14, 7/29/14, 10/20/14 |
| Station Rock | Natural | Pavement & rubble | 34.6 | -77.1 | 15.6 | 9/6/2013, 10/30/13, 6/2/14, 8/20/14, 10/21/14, 5/19/15 |
| Northwest Reef | Natural | Ledge | 34.4 | -76.6 | 21.4 | 8/12/2013, 6/17/14, 10/10/14, 10/20/14, 5/18/15 |
| Southwest of Knuckle Buoy | Natural | Pavement & rubble | 34.4 | -76.5 | 14.2 | 8/20/2013, 10/28/13, 10/20/14, 5/18/15 |
| 10 Fathom | Natural | Ledge | 34.4 | -76.6 | 20.9 | 8/20/2013, 10/29/13, 6/17/14, 8/22/14, 10/20/14, 5/18/15 |
| West Rock | Natural | Pavement & rubble | 34.3 | -76.6 | 24.9 | 9/4/2013, 6/16/14, 10/10/14, 5/27/15 |
| 210 Rock | Natural | Ledge | 34.2 | -76.6 | 30.2 | 9/4/2013, 6/16/14, 10/10/14 |
| Dallas Rocks | Natural | Pavement & rubble | 34.2 | -77.6 | 16.5 | 9/20/2013, 7/1/14, 9/4/14, 11/5/14 |
| 200 / 200 Ledge | Natural | Ledge | 34.1 | -77.4 | 25.1 | 9/20/2013, 11/5/14 |
| 5 Mile Ledge | Natural | Ledge | 34.1 | -77.8 | 15.8 | 9/19/2013, 7/1/14, 9/5/14, 11/13/14 |
| 23 Mile Ledge | Natural | Ledge | 34.0 | -77.4 | 28.7 | 6/24/2014, 9/5/14 |
| Hammerhead Ledge | Natural | Pavement & rubble | 33.5 | -77.9 | 25.4 | 12/13/2013, 6/30/14, 9/11/14, 12/19/14 |
| Thumb Ledge | Natural | Pavement & rubble | 33.5 | -77.9 | 26.5 | 9/25/2013, 6/25/14, 9/11/14 |
| Lightning Bolt Ledge | Natural | Pavement & rubble | 33.5 | -77.9 | 28.5 | 12/13/2013, 6/25/14, 9/11/14, 12/19/14 |
| Bumpy Ledge | Natural | Pavement & rubble | 33.5 | -77.9 | 29.2 | 12/13/2013, 6/25/14, 12/15/14 |

**Table S2:** GLMM model results for A) model including reef type (artificial, natural) as fixed effect and B) model including reef morphology (artificial – ship, artificial – concrete, natural – pavement and rubble, natural – ledge) instead of reef type as fixed effect. For each model, the fixed and random effects are provided. For fixed effects, the χ^2^ and *p*-value from the LRT are provided. For random effects, the variance and standard deviation are listed.

| **Model** | **Effect type** | **Terms** | **χ^2^** | ***p*-value** | **Variance** | **Standard deviation** |
| --- | --- | --- | --- | --- | --- | --- |
| **A)** Reef type | Fixed effects | Reef type | 44.38 | <0.0001 |  |  |
|  |  | Residency | 29.10 | <0.0001 |  |  |
|  |  | Reef type x Residency | 44.42 | <0.0001 |  |  |
|  |  | Depth | 11.15 | <0.001 |  |  |
|  |  | Season | 2.68 | 0.44 |  |  |
|  | Random effects | Reef name |  |  | 0.06 | 0.24 |
|  |  | Sampling event |  |  | 0.15 | 0.39 |
|  |  | Species |  |  | 2.32 | 1.52 |
| **B)** Reef morphology | Fixed effects | Reef morphology | 68.10 | <0.0001 |  |  |
|  |  | Residency | 50.20 | <0.0001 |  |  |
|  |  | Reef morphology x Residency | 68.14 | <0.0001 |  |  |
|  |  | Depth | 12.00 | <0.001 |  |  |
|  |  | Season | 1.89 | 0.60 |  |  |
|  | Random effects | Reef name |  |  | 0.04 | 0.20 |
|  |  | Sampling event |  |  | 0.16 | 0.40 |
|  |  | Species |  |  | 2.33 | 1.53 |

**Table S3:** GLMM predictions of predator density for three depths (m) by reef type, species residency, and sampling season.

| **Depth** | **Reef type** | **Residency** | **Winter** | **Spring** | **Summer** | **Fall** |
| --- | --- | --- | --- | --- | --- | --- |
| 10 | Artificial | Transient | 0.15 ± 0.09 | 0.14 ± 0.08 | 0.20 ± 0.10 | 0.19 ± 0.10 |
|  |  | Resident | 0.09 ± 0.05 | 0.08 ± 0.04 | 0.12 ± 0.05 | 0.11 ± 0.05 |
|  | Natural | Transient | 0.04 ± 0.02 | 0.04 ± 0.02 | 0.05 ± 0.03 | 0.05 ± 0.03 |
|  |  | Resident | 0.07 ± 0.04 | 0.06 ± 0.03 | 0.09 ± 0.04 | 0.09 ± 0.04 |
| 20 | Artificial | Transient | 0.24 ± 0.13 | 0.22 ± 0.12 | 0.31 ± 0.15 | 0.31 ± 0.15 |
|  |  | Resident | 0.14 ± 0.07 | 0.13 ± 0.06 | 0.18 ± 0.08 | 0.18 ± 0.08 |
|  | Natural | Transient | 0.06 ± 0.04 | 0.06 ± 0.03 | 0.08 ± 0.04 | 0.08 ± 0.04 |
|  |  | Resident | 0.11 ± 0.06 | 0.10 ± 0.05 | 0.14 ± 0.06 | 0.14 ± 0.06 |
| 30 | Artificial | Transient | 0.38 ± 0.21 | 0.35 ± 0.19 | 0.50 ± 0.25 | 0.48 ± 0.24 |
|  |  | Resident | 0.22 ± 0.12 | 0.20 ± 0.11 | 0.29 ± 0.13 | 0.28 ± 0.13 |
|  | Natural | Transient | 0.10 ± 0.06 | 0.09 ± 0.05 | 0.13 ± 0.07 | 0.13 ± 0.07 |
|  |  | Resident | 0.18 ± 0.09 | 0.16 ± 0.08 | 0.23 ± 0.11 | 0.22 ± 0.10 |

**Table S4:** GLMM predictions of predator density for three depths (m) by reef morphology, species residency, and sampling season.

| **Depth** | **Reef morphology** | **Residency** | | **Winter** | **Spring** | **Summer** | **Fall** |
| --- | --- | --- | --- | --- | --- | --- | --- |
| 10 | Ship | Transient | 0.19 ± 0.11 | | 0.16 ± 0.09 | 0.23 ± 0.12 | 0.22 ± 0.11 |
|  |  | Resident | 0.08 ± 0.04 | | 0.07 ± 0.04 | 0.10 ± 0.05 | 0.09 ± 0.04 |
|  | Concrete | Transient | 0.08 ± 0.05 | | 0.07 ± 0.04 | 0.10 ± 0.06 | 0.10 ± 0.05 |
|  |  | Resident | 0.13 ± 0.08 | | 0.11 ± 0.06 | 0.16 ± 0.08 | 0.15 ± 0.08 |
|  | Ledge | Transient | 0.05 ± 0.03 | | 0.05 ± 0.03 | 0.06 ± 0.03 | 0.06 ± 0.03 |
|  |  | Resident | 0.09 ± 0.05 | | 0.07 ± 0.04 | 0.10 ± 0.05 | 0.10 ± 0.05 |
|  | Pavement & rubble | Transient | 0.03 ± 0.02 | | 0.03 ± 0.02 | 0.04 ± 0.02 | 0.04 ± 0.02 |
|  |  | Resident | 0.06 ± 0.04 | | 0.05 ± 0.03 | 0.08 ± 0.04 | 0.07 ± 0.04 |
| 20 | Ship | Transient | 0.31 ± 0.17 | | 0.26 ± 0.14 | 0.37 ± 0.18 | 0.36 ± 0.18 |
|  |  | Resident | 0.13 ± 0.07 | | 0.11 ± 0.06 | 0.16 ± 0.07 | 0.15 ± 0.07 |
|  | Concrete | Transient | 0.14 ± 0.08 | | 0.12 ± 0.07 | 0.16 ± 0.09 | 0.16 ± 0.09 |
|  |  | Resident | 0.22 ± 0.12 | | 0.18 ± 0.10 | 0.26 ± 0.12 | 0.25 ± 0.12 |
|  | Ledge | Transient | 0.09 ± 0.05 | | 0.07 ± 0.04 | 0.10 ± 0.05 | 0.10 ± 0.05 |
|  |  | Resident | 0.14 ± 0.08 | | 0.12 ± 0.06 | 0.17 ± 0.08 | 0.16 ± 0.08 |
|  | Pavement & rubble | Transient | 0.06 ± 0.03 | | 0.05 ± 0.03 | 0.07 ± 0.04 | 0.07 ± 0.03 |
|  |  | Resident | 0.1 ± 0.050 | | 0.09 ± 0.05 | 0.12 ± 0.06 | 0.12 ± 0.06 |
| 30 | Ship | Transient | 0.50 ± 0.28 | | 0.43 ± 0.24 | 0.59 ± 0.30 | 0.58 ± 0.29 |
|  |  | Resident | 0.21 ± 0.11 | | 0.18 ± 0.09 | 0.25 ± 0.12 | 0.24 ± 0.11 |
|  | Concrete | Transient | 0.22 ± 0.14 | | 0.19 ± 0.11 | 0.26 ± 0.15 | 0.25 ± 0.15 |
|  |  | Resident | 0.35 ± 0.20 | | 0.30 ± 0.16 | 0.42 ± 0.21 | 0.41 ± 0.21 |
|  | Ledge | Transient | 0.14 ± 0.08 | | 0.12 ± 0.07 | 0.16 ± 0.09 | 0.16 ± 0.09 |
|  |  | Resident | 0.23 ± 0.12 | | 0.20 ± 0.10 | 0.27 ± 0.13 | 0.27 ± 0.13 |
|  | Pavement & rubble | Transient | 0.09 ± 0.05 | | 0.08 ± 0.05 | 0.11 ± 0.06 | 0.11 ± 0.06 |
|  |  | Resident | 0.17 ± 0.08 | | 0.14 ± 0.07 | 0.20 ± 0.09 | 0.19 ± 0.09 |

**Table S5: Studies from which data were extracted during the literature synthesis.** For each study, the author, date, and title are provided. The metric reported for fish is also provided.

| **Author** | **Date** | **Title** | **Metric** |
| --- | --- | --- | --- |
| Hackradt et al. | 2011 | Influence of habitat structure on fish assemblage of an artificial reef in southern Brazil | abundance |
| Oakes & Pondella | 2009 | The value of net-cage as a fish aggregating device in southern California | density |
| Granneman & Steele | 2015 | Effects of reef attributes on fish assemblage similarity between artificial and natural reefs | density |
| Simon et al. | 2013 | Fish assemblages on shipwrecks and natural rocky reefs strongly differ in trophic structure | biomass |
| Santos et al. | 2013 | A comparison of the fish assemblages on natural and artificial reefs off Sal Island (Cape Verde) | density |
| Bryan et al. | 2013 | Characterization of the mesophotic reef fish community in south Florida, USA | abundance |

**Table S6: Species data extracted during the literature synthesis.** For each species, the family, scientific name, and common name, and trophic level according to Fishbase are provided. The habitat type (natural or artificial) where the top predators exhibited higher values of abundance, biomass, or density is given, as is the study from which these data were extracted, referenced in Table S2.

| **Family** | **Scientific Name** | **Common Name** | **Trophic Level** | **Habitat Type** | **Residency** | **Study** |
| --- | --- | --- | --- | --- | --- | --- |
| Carangidae | Seriola dumerili | Greater Amberjack | 4.5 | Artificial | Transient | Bryan-et-al-2013 |
|  | Seriola dumerili | Greater Amberjack | 4.5 | Natural | Transient | Santos-et-al-2013 |
|  | Seriola rivoliana | Almaco Jack | 4.5 | Artificial | Transient | Bryan-et-al-2013 |
|  | Seriola rivoliana | Almaco Jack | 4.5 | Artificial | Transient | Hackradt-et-al-2011 |
|  | Seriola rivoliana | Almaco Jack | 4.5 | Artificial | Transient | Santos-et-al-2013 |
| Hexagrammidae | Ophiodon elongatus | Lingcod | 4.5 | Artificial | Resident | Granneman-&-Steele-2015 |
| Lutjanidae | Lutjanus cyanopterus | Cubera Snapper | 4.4 | Artificial | Resident | Bryan-et-al-2013 |
|  | Lutjanus jocu | Dog Snapper | 4.4 | Artificial | Resident | Simon-et-al-2013 |
| Muraenidae | Gymnothorax mordax | California Moray | 4.5 | Artificial | Resident | Granneman-&-Steele-2015 |
|  | Gymnothorax moringa | Spotted Moray | 4.5 | Natural | Resident | Hackradt-et-al-2011 |
|  | Gymnothorax moringa | Spotted Moray | 4.5 | Natural | Resident | Santos-et-al-2013 |
|  | Gymnothorax moringa | Spotted Moray | 4.5 | Artificial | Resident | Simon-et-al-2013 |
| Paralichthyidae | Paralichthys brasiliensis | Brazilian Flounder | 4.4 | Natural | Resident | Simon-et-al-2013 |
|  | Paralichthys californicus | California Flounder | 4.5 | Artificial | Resident | Granneman-&-Steele-2015 |
| Polyprionidae | Stereolepis gigas | Giant Seabass | 4.5 | Natural | Resident | Granneman-&-Steele-2015 |
| Pomatomidae | Pomatomus saltatrix | Bluefish | 4.5 | Artificial | Transient | Hackradt-et-al-2011 |
| Scombridae | Scomberomorus cavalla | King Mackerel | 4.4 | Artificial | Transient | Hackradt-et-al-2011 |
|  | Scomberomorus sierra | Pacific Sierra | 4.5 | Artificial | Transient | Oakes-&-Pondella-2009 |
| Serranidae | Mycteroperca interstitialis | Yellowmouth Grouper | 4.5 | Artificial | Resident | Bryan-et-al-2013 |
|  | Mycteroperca interstitialis | Yellowmouth Grouper | 4.5 | Natural | Resident | Hackradt-et-al-2011 |
|  | Mycteroperca interstitialis | Yellowmouth Grouper | 4.5 | Artificial | Resident | Simon-et-al-2013 |
|  | Mycteroperca phenax | Scamp | 4.5 | Artificial | Resident | Bryan-et-al-2013 |
| Sphyraenidae | Sphyraena argentea | Pacific Barracuda | 4.5 | Natural | Transient | Granneman-&-Steele-2015 |
|  | Sphyraena argentea | Pacific Barracuda | 4.5 | Natural | Transient | Oakes-&-Pondella-2009 |
|  | Sphyraena barracuda | Great Barracuda | 4.5 | Artificial | Transient | Bryan-et-al-2013 |
|  | Sphyraena guachancho | Guachanche Barracuda | 4.4 | Artificial | Transient | Hackradt-et-al-2011 |
